# Functional dysconnectivity in Schizophrenia and Bipolar Disorder: associations with cognitive impairment

**DOI:** 10.1192/j.eurpsy.2025.103

**Published:** 2025-08-26

**Authors:** G. Cattarinussi

**Affiliations:** 1 Department of Psychological Medicine, King’s College London, London, United Kingdom; 2 Department of Neuroscience, University of Padova, Padova, Italy

## Abstract

**Background:**

Abnormal cerebellar functional connectivity (FC) has been independently implicated in the pathophysiology of schizophrenia (SCZ) and bipolar disorder (BD). However, the relationship between cerebellar dysconnectivity patterns in these two disorders and their association with cognitive functioning and clinical symptoms have not been fully clarified. In this study, we used the state-of-the-art functional atlas of the cerebellum to examine cerebellar FC changes in the SCZ–BD spectrum and their association with cognitive and clinical variables.

**Methods:**

Resting-state functional magnetic resonance imaging (fMRI) data of 39 individuals with SCZ, 43 BD type I and 61 healthy controls were examined. The cerebellum was parcellated into ten functional systems and we calculated seed-based FC for each cerebellar system. Cognitive abilities were investigated with the Wechsler memory scale, the California Verbal Learning test, the Stroop test, the Attentional network task, the Continuous performance test, the Task Switch task and the Stop Signal task. Psychopathological evaluation was carried out using the Scale for the Assessment of Negative Symptoms and the Scale for the Assessment of Positive Symptoms. We used principal component analyses to reduce the dimensionality of the diagnosis-related FC and cognitive variables, respectively. Multiple regression analyses were conducted to assess the relationship between FC and cognitive and clinical data.

**Results:**

We observed lower cerebellar FC with the frontal, temporal, occipital and thalamic areas in SCZ, and a more widespread decrease in cerebellar FC in BD, involving the frontal, cingulate, parietal, temporal, occipital and thalamic regions. SCZ presented increased within-cerebellum and cerebellar-frontal FC compared to BD. Higher cortico-cerebellar FC was positively associated with memory (p-FWE=0.036) and verbal learning (p-FWE=0.043). Exploratory analyses showed a negative correlation between cortico-cerebellar FC and positive symptoms (p-FWE=0.051).

**Conclusions:**

These findings suggest a role for shared and distinct patterns of corticocerebellar dysconnectivity in the SCZ–BD spectrum that can result in cognitive impairment and psychotic symptoms. In addition, they highlight the potential role of cerebellar stimulation as a promising intervention for individuals with SCZ and BD-I that present cognitive impairment.

**Disclosure of Interest:**

None Declared

